# Phenotypic Features of Circulating Leukocytes from Non-human Primates Naturally Infected with *Trypanosoma cruzi* Resemble the Major Immunological Findings Observed in Human Chagas Disease

**DOI:** 10.1371/journal.pntd.0004302

**Published:** 2016-01-25

**Authors:** Renato Sathler-Avelar, Danielle Marquete Vitelli-Avelar, Armanda Moreira Mattoso-Barbosa, Marcelo Perdigão-de-Oliveira, Ronaldo Peres Costa, Silvana Maria Elói-Santos, Matheus de Souza Gomes, Laurence Rodrigues do Amaral, Andréa Teixeira-Carvalho, Olindo Assis Martins-Filho, Edward J. Dick, Gene B. Hubbard, Jane F. VandeBerg, John L. VandeBerg

**Affiliations:** 1 Grupo Integrado de Pesquisas em Biomarcadores, Centro de Pesquisas René Rachou, Fundação Oswaldo Cruz-FIOCRUZ, Belo Horizonte, Minas Gerais, Brazil; 2 Centro Universitário Newton Paiva, Belo Horizonte, Minas Gerais, Brazil; 3 Pós-graduação em Patologia, Faculdade de Medicina, UFMG, Belo Horizonte, Minas Gerais, Brazil; 4 Texas Biomedical Research Institute, San Antonio, Texas, United States of America; 5 Pós-graduação em Ciências da Saúde, Centro de Pesquisas René Rachou, FIOCRUZ, Belo Horizonte, Minas Gerais, Brazil; 6 Departamento de Propedêutica Complementar, Faculdade de Medicina, Universidade Federal de Minas Gerais, Belo Horizonte, Minas Gerais, Brazil; 7 Laboratório de Bioinformática e Análise Molecular, Instituto de Genética e Bioquímica Universidade Federal de Uberlândia, Campus Patos de Minas, Patos de Minas, Minas Gerais, Brazil; 8 Laboratório de Bioinformática e Análise Molecular, Faculdade de Ciência da Computação, Universidade Federal de Uberlândia, Campus Patos de Minas, Patos de Minas, Minas Gerais, Brazil; 9 South Texas Diabetes and Obesity Institute, University of Texas Health Science Center, San Antonio – Regional Academic Health Center, Edinburg, Texas, United States of America; Institute of Tropical Medicine (NEKKEN), JAPAN

## Abstract

**Background:**

Cynomolgus macaques (*Macaca fascicularis*) represent a feasible model for research on Chagas disease since natural *T*. *cruzi* infection in these primates leads to clinical outcomes similar to those observed in humans. However, it is still unknown whether these clinical similarities are accompanied by equivalent immunological characteristics in the two species. We have performed a detailed immunophenotypic analysis of circulating leukocytes together with systems biology approaches from 15 cynomolgus macaques naturally infected with *T*. *cruzi* (CH) presenting the chronic phase of Chagas disease to identify biomarkers that might be useful for clinical investigations.

**Methods and Findings:**

Our data established that CH displayed increased expression of CD32^+^ and CD56^+^ in monocytes and enhanced frequency of NK Granzyme A^+^ cells as compared to non-infected controls (NI). Moreover, higher expression of CD54 and HLA-DR by T-cells, especially within the CD8^+^ subset, was the hallmark of CH. A high level of expression of Granzyme A and Perforin underscored the enhanced cytotoxicity-linked pattern of CD8^+^ T-lymphocytes from CH. Increased frequency of B-cells with up-regulated expression of Fc-γRII was also observed in CH. Complex and imbricate biomarker networks demonstrated that CH showed a shift towards cross-talk among cells of the adaptive immune system. Systems biology analysis further established monocytes and NK-cell phenotypes and the T-cell activation status, along with the Granzyme A expression by CD8^+^ T-cells, as the most reliable biomarkers of potential use for clinical applications.

**Conclusions:**

Altogether, these findings demonstrated that the similarities in phenotypic features of circulating leukocytes observed in cynomolgus macaques and humans infected with *T*. *cruzi* further supports the use of these monkeys in preclinical toxicology and pharmacology studies applied to development and testing of new drugs for Chagas disease.

## Author Summary

*T*. *cruzi* is the parasite responsible for Chagas disease, a neglected tropical illness, present in endemic and also in non-endemic countries. *T*. *cruzi* parasites are spread mainly by a vector’s bite but can also be transmitted by blood transfusion, organ transplant, laboratory accidents, congenitally and by ingestion of contaminated food. Non-human primates that are also predisposed to become infected, live in places where vectors and *T*. *cruzi* exist. Similar clinical sequelae are observed in these animals when compared to humans who are infected with *T*. *cruzi*. A better understanding of the pathogenesis of *T*. *cruzi*-infected non-human primates may bring new advances to understanding human infection. Here, we explored the immunological features of cynomolgus macaques naturally infected by *T*. *cruzi*, aiming to contribute to the validation of this species as an appropriate experimental model. Infected animals displayed a similar immunological profile to that observed in humans, with high activity of cytotoxic cells and expansion of macrophages and T-cell subsets. Furthermore, by using bioinformatics tools, we demonstrated that CD14^+^CD56^+^ and CD3^+^HLA-DR^+^ cells are major determinants to segregate CH from NI group, followed by innate and adaptive cell subpopulations. Altogether, our data suggest that non-human primates are an appropriate model to study Chagas disease.

## Introduction

The haemoflagellate *Trypanosoma cruzi* causes Chagas disease, one of the most important neglected tropical diseases of humankind [1]. There are currently an estimated 6 million to 7 million people infected worldwide, predominantly in Latin America, where infection with *T*. *cruzi* is endemic, and more than 25 million people are at risk of becoming infected [2]. Nevertheless, non-endemic areas are also at risk of an increasing health curve burden of Chagas disease, mainly due to the high level of emigration from endemic to developed countries [3].

*T*. *cruzi* infection usually progresses from an acute infection to a chronic disease characterized by low, but persistent parasitism, accompanied by a complex host–parasite relationship and imbricate activation and modulation of immunological events [4]. Besides the relevance of the immune system to the development and maintenance of different clinical forms of Chagas disease [4], immunological events seem to be associated with the therapeutic efficacy of benznidazole [5,6], which is the drug of choice for treating Chagas disease. Despite the rapid advances in Chagas disease research from basic research, further investigation is required to decipher several parasite-host interaction mechanisms in order to support the rational proposal of novel diagnostic strategies, supportive clinical monitoring tools, the discovery of new drugs, and the establishment of combined multi-drug therapeutic protocols.

In the field of drug development, the validation of experimental models is essential for enabling valid pre-clinical trials. Although murine and canine experimental models have been used for research on Chagas disease, in regard both to clinical disease manifestation and pre-clinical drug testing [7,8,9], particular physiological features of these mammalian hosts suggest that other models more closely related to humans are required for pre-clinical trials to ensure validity of translation of results to the human condition.

Several non-human primates are predisposed to get naturally infected by *T*. *cruzi* and develop similar clinical outcomes to those observed in human Chagas disease [10,11]. There have been reports of natural infection of *T*. *cruzi* in cynomolgus macaques (*Macaca fascicularis*), and the development of cardiomyopathy consistent with Chagas disease supporting the notion that these Old World non-human primates can manifest similar clinical disorder as observed in human Chagas disease [11,12]. Cynomolgus macaques are an important non-human primate in biomedical research and are widely used in drug development, drug testing, and toxicology. In addition to their small body size, the similarities to humans in physiological features and susceptibility to infectious diseases make cynomolgus macaques as experimental models for Chagas disease pre-clinical investigations and drug trials. To date, despite several studies that have been conducted with non-human primates infected with *T*. *cruzi* [11,12], the detailed immunological events triggered by the *T*. *cruzi* infection in any non-human primate remain to be elucidated. The investigation reported here has applied a systems biology approach to bring insights that improve our comprehension of the immunological aspects of *T*. *cruzi* infection in the cynomolgus macaque model. Cytomics represents an innovative tool of systems biology that aim to determine the molecular phenotype at the single cell level and further represent its neighborhood connections in cellular systems [13,14]. Conventional and multi-color fluorescence-based flow cytometry at the single-cell level, associated with bioinformatics software, has become an important tool in cytomics systems biology, and we have used it for analyses that link the dynamics of cell phenotype and function at high content and high throughput.

In this study we have performed a detailed single-cell phenotypic analysis of peripheral blood leucocytes and applied conventional and systems biology approaches to evaluate the immunological features of cynomolgus macaques naturally infected with *T*. *cruzi*, aiming to identify putative biomarkers that have similarities to those of humans infected with *T*. *cruzi*. Our findings provide further data to validate cynomolgus macaques as a model for pre-clinical studies of Chagas disease.

## Material and Methods

### Study Population

The experiments were carried out with 26 cynomolgus macaques consisting of 21 females and five males. All subjects were submitted to serological screening tests to detect anti-*T*. *cruzi* antibodies by enzyme-linked immunoassay (ELISA; Bio-Manguinhos; Oswaldo Cruz Foundation, Rio de Janeiro, Brazil) and immuno-chromatographic assay (Chagas STATPAK; Chembio Diagnostic Systems, Medford, NY). Based on the serological status, the primates were segregated into two groups, referred to as: *T*. *cruzi* naturally infected primates (CH), presenting positive serology in both tests, comprising 12 females and three males (median age = 12 years, age ranging from 2–20 years; median weight = 3.5kg, ranging from 1.9–7.9 kg); and non-infected controls (NI), including nine females and two males (median age = 13 years, age ranging from 1–20 years; median weight = 4.9kg, ranging from 1.9–7.6 kg), presenting negative serology in both tests. All *T*. *cruzi*-naturally infected cynomolgus enrolled in the present investigation presented the indeterminate chronic phase of Chagas disease, defined by the absence of patent parasitemia characteristic of chronic Chagas disease and by meticulous organ inspections carried out during necropsy to access the macroscopic aspects of esophagus, colon and heart. The gastrointestinal tract did not present any macroscopic sign of megaesophagus or megacolon, suggestive of digestive clinical form of Chagas disease. Moreover, the myocardium of all animals presented a macroscopically normal aspect, without signs of wall aneurysms. Moreover, the volume and the weight of all hearts were within normal limits. Together, these features fulfilled the criterion described by Dias et al. [15].

### Ethics Statement

The cynomolgus macaques (*Macaca fascicularis*) included in this cross sectional study were housed in metal and concrete indoor/outdoor enclosures at the Southwest National Primate Research Center (SNPRC), San Antonio, TX, USA. The macaques were provided water and food *ad libitum*, the food consisting of commercial monkey chow, vegetables and fruits. The animals were maintained in accordance with the Guide for the Care and Use of Laboratory Animals under protocols approved by the Institutional Animal Care and Use Committee (#1050MF). This study was conducted in accordance with the U.S Animal Welfare Act, and the Public Health Service Policy on Humane Care and Use of Laboratory Animals.

### Blood Samples

General anesthesia was achieved by immobilizing the animals with an intramuscular injection of ketamine hydrochloride (10mg/kg) and valium (5mg). Besides that, the animals were kept up on isofluorane (1.5%) inhalation. Following anesthesia, 5mL sample of peripheral blood was collected from the femoral vein of each animal using ethylenediamine tetraacetic acid (EDTA) as the anticoagulant. After blood collection, the immunophenotypic features of peripheral blood leucocytes were analyzed by flow cytometry.

### Monoclonal Antibodies Used for Immunophenotyping

Mouse anti-human monoclonal antibodies (mAbs) specific for cell surface markers, showing cross-reactivity to non-human primates, were used in this study. Multiparametric flow cytometry immunophenotyping approaches were carried out by simultaneous use of fluorescein isothiocyanate-FITC, phycoerythrin-PE, PerCP-Cy5.5, APC or Alexa fluor 700 conjugated mAbs. The panels were assembled with anti-CD4 (L200), anti-CD14 (322A-1), anti-CD16 (3G8), anti-CD32 (FLI.826), anti-CD64 (10.1), anti-Granzyme A (CB9), anti-Granzyme B (GB11) and anti-Perforin (DG9) antibodies labeled with FITC; anti-CD4 (L200), anti-CD14 (MϕP9), anti-CD54 (LB-2), anti-CD56 (B159) and anti-CD69 (FN50) antibodies labeled with PE; anti-CD4 (L200), anti-CD8 (SK1) and anti-HLA-DR (L243) antibodies conjugated with PerCP-Cy5.5; anti-CD8 (3B5), anti-CD16 (3G8) and anti-CD20 (2H7) antibodies conjugated with APC and anti-CD3 (SP34-2) antibodies conjugated with Alexa fluor 700. Fluorescent labeled mouse isotypic reagents were included as internal controls in all flow cytometric batches.

### Immunostaining for Cell Surface Markers

Whole blood cell samples were used for immunophenotypic analysis as recommended by the monoclonal antibody manufacturer, Becton- Dickinson (Mountain View, CA, USA), modified as follows: 100μL of whole peripheral blood was incubated with 5μL of undiluted fluorescent labeled mAb in 12x75mm tubes in the dark, for 30 min at room temperature. Following incubation, lysis of erythrocytes was performed by the addition of 2mL of FACS Lysing Solution (Becton Dickinson Biosciences Pharmingen, San Diego, CA, USA) vortexing, followed by incubation in the dark for 10 min at room temperature. The leukocyte suspension was then washed twice with phosphate-buffered saline (PBS) containing 0.01% sodium azide. Stained cells were fixed with 200μL of FACS-FIX Solution (10g/L paraformaldehyde, 10.2g/L sodium cacodylate, 6.65 g/L sodium-chloride, 0.01% sodium azide) and stored at 4°C, up to 24h, until flow cytometry processing.

### Intracellular Cytotoxicity Markers Staining of CD16^+^ and CD8^+^ Cells

Intracellular analyses of Granzyme A, Granzyme B and Perforin in CD16^+^ and CD8^+^ cells were performed by staining 100μL of whole blood with 5μL of anti-CD16 or anti-CD8 mAbs, in the dark for 30 min at room temperature. Following incubation, erythrocytes were lysed and leukocytes were fixed with FACS-FIX Solution, and the remaining cell suspension was permeabilized with 2mL of FACS perm-buffer (FACS buffer supplemented with 0.5% saponin, Sigma), in the dark for 10 min at room temperature. Following, cells were washed with 2mL and resuspended into 100μL FACS perm-buffer. The fixed/permeabilized, membrane-stained leukocyte suspension was then incubated with 5μL of anti-Granzyme A, anti-Granzyme B or anti-Perforin in the dark, for 30 min at room temperature. After intracytoplasmic staining, the cells were washed once with FACS perm-buffer, followed by one wash with FACS buffer and then fixed in 200μL of FACS-FIX Solution and stored at 4°C, up to 24h, until flow cytometry processing.

### Flow Cytometry Acquisition and Analyses

A total of 30,000 events per sample were acquired in a CyAn ADP flow cytometry analyzer (Beckman Coulter, Inc., Brea, CA, USA). Data acquisition and analyses were performed using the Summit software 4.3.01 (Beckman Coulter, Inc., Brea, CA, USA). Distinct gate strategies, as previously described by Vitelli-Avelar et al. [16], were applied for data analysis using the FlowJo software (version 9.4.1, TreeStar Inc. Ashland, OR, USA).

### Data Analysis

#### Conventional statistics

Comparative analysis between groups was performed by non- parametric Mann Whitney test, using GraphPad Prism software (version 5.03, San Diego, California, USA). Additional analysis was performed by Spearman’s correlation test. Significance was set at *p<*0.05.

#### Biomarker networks assembling

Biomarker networks were assembled to assess the association between cell subpopulations (monocytes, NK cells, T cells and B cells) and their subsets for each clinical group. Significant correlations representing the interaction between biomarkers tested were compiled using the open source software Cytoscape (version 3.1.1), as previously reported [17]. The biomarker networks were constructed using circle layouts with each biomarker represented by specific globular nodes (NI = white nodes; CH = black nodes). Connecting edges represent correlation scores categorized as positive strong (r ≥ 0.68; thick black line), positive moderate (0.36 ≤ r < 0.68; thin black line), negative strong (r ≤ -0.68; thick gray dotted line), negative moderate (-0.68 < r ≤ -0.36; thin gray dashed line) as proposed by Taylor [18].

#### Heatmaps and decision tree analysis

The heatmaps were produced using the heatmap.2 function in the R (Project for Statistical Computing Version 3.0.1) and gplots package. All analyses were performed using customized functions available from Bioconductor packages. After dataset analysis, a decision tree was generated for each heat map. The decision trees, the most widely used machine learning algorithms, were used to select the minimal set of phenotypic features that efficiently segregated groups. The decision tree was built using the WEKA software (Waikato Environment for Knowledge Analysis, version 3.6.11, University of Waikato, New Zealand). This method analyzes all the phenotypic attributes in the training set and selects the most relevant attribute that maximizes the information gain as the root node. Following, the method continues searching for additional attributes for group segregation. In order to estimate the classification accuracy of the decision tree models on new data with unknown class labels, a 10-fold cross validation methodology available in the WEKA software was applied.

## Results

### Increased Expression of CD32^+^ and CD56^+^ Activation Markers on Monocytes of *T*. *cruzi*-Infected Monkeys

**Fig 1:**
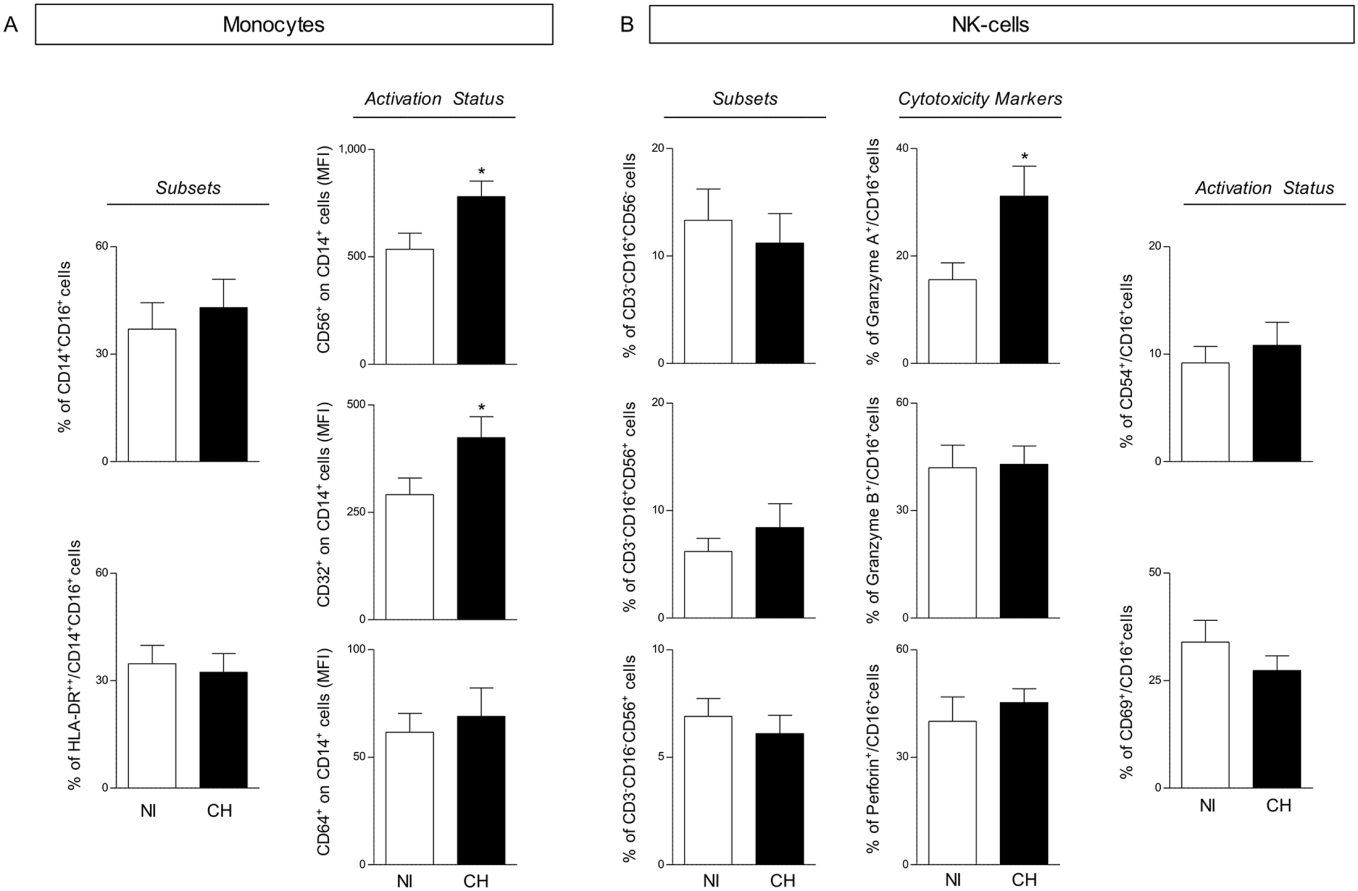
Innate immunity features from cynomolgus macaques naturally infected with *T*. *cruzi* (CH) and non-infected controls (NI). (A) Flow cytometry immunophenotyping platforms were assembled to quantify the percentage of macrophage-like (CD14^+^CD16^+^) and pro-inflammatory (CD14^+^CD16^+^HLA-DR^++^) events within gated monocytes. Activation status was estimated by the analysis of CD56 and FcγR (CD32, CD64) expression by circulating monocytes, and data are reported as the Mean Fluorescence Intensity (MFI). (B) Analyses of NK-cells were performed to quantify NK subpopulations (CD3^-^CD16^+^CD56^-^, CD3^-^CD16^+^CD56^+^ and CD3^-^CD16^-^CD56^+^), the cytotoxicity profile (Granzyme A, Granzyme B and Perforin) and activation markers (CD69 and CD54). The results are expressed as mean percentage with standard error. Significant differences at *p<*0.05 are identified by asterisks (*).

The analyses of monocyte subsets and activation status are shown in Fig 1A. Data analyses revealed that the frequency of CD14^+^CD16^+^ macrophage-like cells and CD14^+^ HLA-DR^++^ pro-inflammatory monocytes subsets did not differ between groups. However, despite the unaltered expression of CD64, monocytes from *T*. *cruzi*-infected monkeys showed increased expression of activation-related surface markers, such as CD32 and CD56 (Fig 1A).

### Enhanced Frequency of NK Granzyme A^+^ Cells Are Observed in *T*. *cruzi*-Infected Monkeys

The frequency of NK-cell subsets, along with the intracytoplasmic expression of cytotoxicity-linked molecules and activation-related surface markers are shown in Fig 1B. Despite no difference in the frequency of circulating NK-cell subsets, a higher percentage of NK Granzyme A^+^ cells was observed in *T*. *cruzi*-infected monkeys as compared to non-infected monkeys. No differences in the percentage of Granzyme B^+^ and Perforin^+^, or in CD69^+^ or CD54^+^ NK cells, were observed between groups (Fig 1B).

### Higher Expression of Adhesion/Activation Molecules by T-Lymphocytes, Particularly CD54 and HLA-DR within the CD8^+^ T-Cell Subset, Are the Hallmarks of *T*. *cruzi*-Infected Monkeys

**Fig 2:**
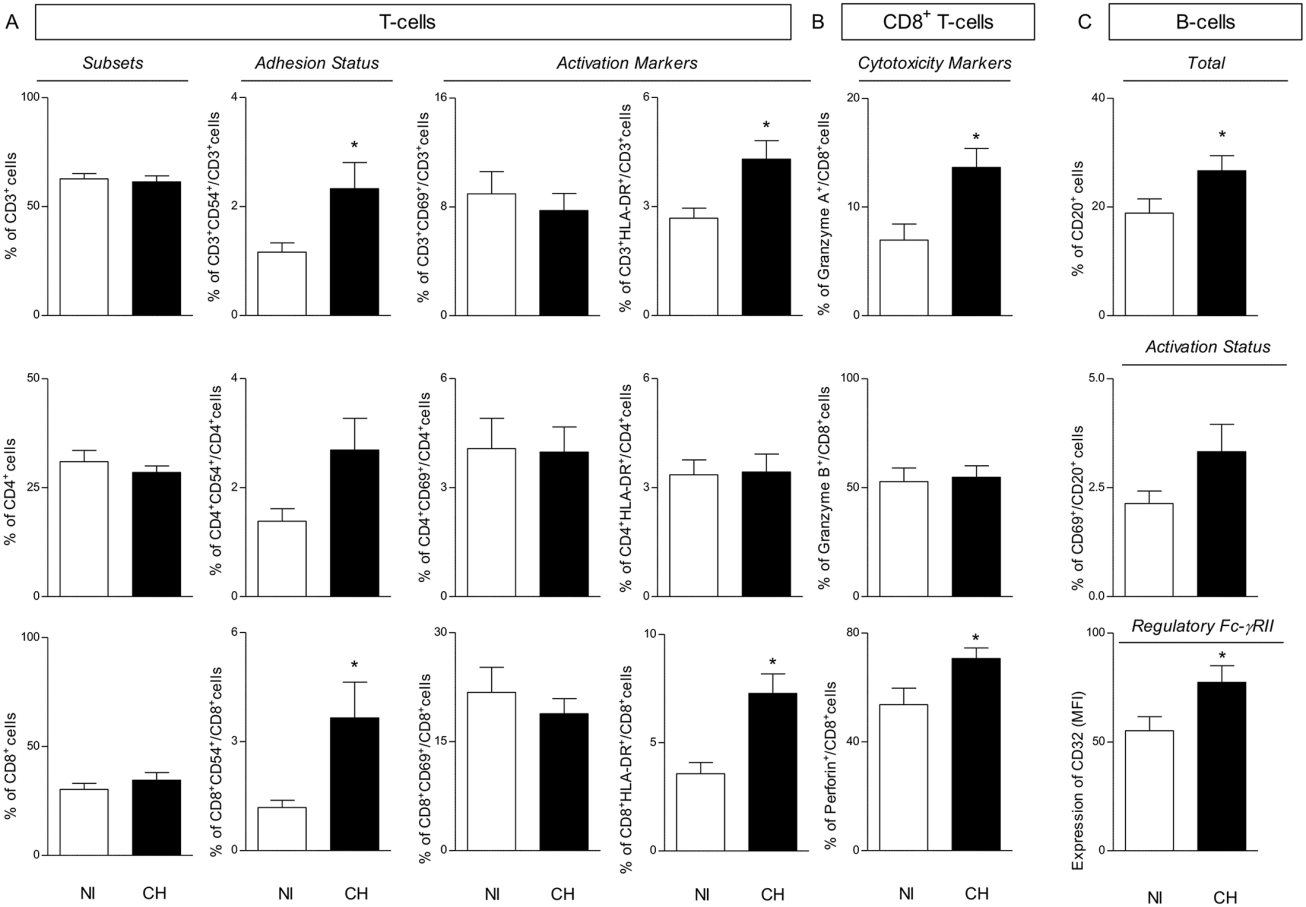
Adaptive immunity features from cynomolgus macaques naturally infected with *T*. *cruzi* (CH) and non-infected controls (NI). (A) The frequencies of CD3^+^ lymphocytes and T-cell subsets (CD4^+^ and CD8^+^), the expression of adhesion molecule (CD54) and activation status (CD69 and HLADR) were performed by multicolor flow cytometry. (B) The expression of cytotoxicity markers (Granzyme A, Granzyme B and Perforin) of CD8^+^ T-cells was investigated by intracellular staining flow cytometry. (C) Analysis of B-cells, the activation status (CD69), and the expression of the regulatory FcγR (CD32) were evaluated by three-color flow cytometry. The results are expressed as mean percentage with standard error. Significant differences at *p<*0.05 are identified by (*).

The analyses of T-cell subsets, adhesion molecule expression and activation status are shown in Fig 2A. Although no differences were observed in the frequency of circulating T-lymphocytes and CD4^+^ or CD8^+^ T-cell subsets, higher percentages of CD3^+^CD54^+^ cells and CD8^+^CD54^+^ T-cells were found in infected monkeys than in non-infected monkeys (Fig 2A). The analysis of activation-related markers revealed an increased percentage of HLA-DR^+^ T-cells, selectively within the CD8^+^ T-cell subset, in infected monkeys, with no difference in the percentage of early activated CD69^+^ T-cells. No significant differences were found in adhesion molecule expression or activation status of the circulating CD4^+^ T-cell subset (Fig 2A).

### Increased Expression of Granzyme A and Perforin Intracytoplasmic Markers Underscore the Enhanced Cytotoxicity-Linked Pattern of Circulating CD8+ T-Lymphocytes from Infected Primates

Additional analyses of cytotoxicity-linked intracytoplasmic marker expression by CD8^+^ T-lymphocytes are shown in Fig 2B. The results revealed an increased frequency of Granzyme A^+^ as well as Perforin^+^ CD8^+^ T-cells in infected monkeys than in non-infected monkeys. No difference was observed in the expression of Granzyme B by CD8^+^ T-lymphocytes (Fig 2B).

### Increased Frequency of B-Cells with Up-Regulated Expression of Fc-γRII Is Found in *T. cruzi*-Infected Primates

The percentage of peripheral blood B-cells along with their activation/regulatory status are shown in Fig 2C. The statistical analysis demonstrated that despite the increased percentage of circulating B-cells observed in infected primates, no significant difference in their early activation status (CD69) was found in *T*. *cruzi*-infected as compared to non-infected monkeys. Interestingly, up-regulated expression of the regulatory cell surface molecule CD32 by B-cells was characteristic of infected monkeys as compared to non-infected controls (Fig 2C).

### Complex and Imbricate Biomarker Network Underscores the shift Towards Cross-Talk among Adaptive Immunity Cells in Infected Primates

**Fig 3:**
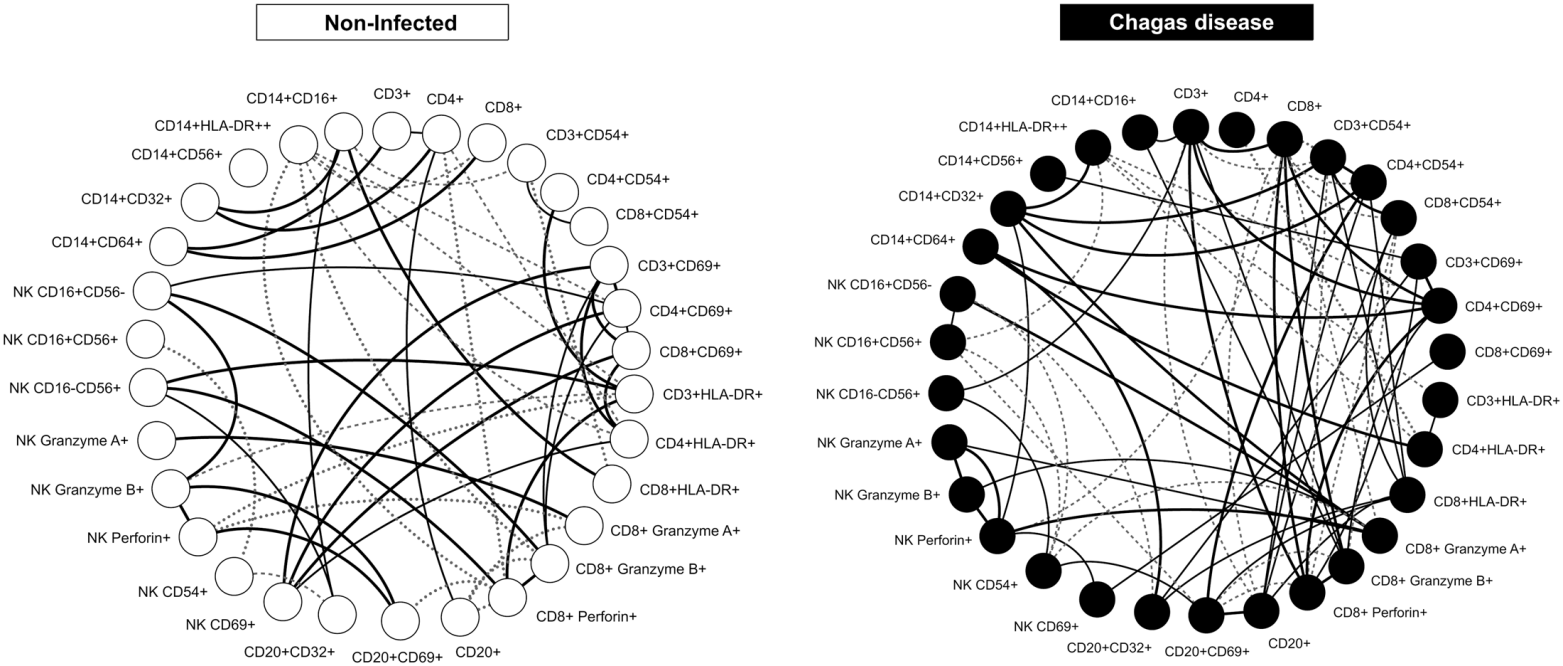
Biomarker networks of immune system from cynomolgus macaques naturally infected with *T*. *cruzi* (CH) and non-infected controls (NI). Circular layouts were built to underscore the relevant associations between cell subsets, the expression of adhesion molecules, cytotoxicity markers, and the activation/regulatory status, using a clustered distribution of nodes for innate (left side) and adaptive (right side) immunity cells. The overall statistical analysis of the network node neighborhood connections points out to a uniform pattern in non-infected controls and a clear shift towards a bimodal profile in *T*. *cruzi*-infected hosts, with prominent involvement of adaptive immunity events.

Exploratory analysis of biomarker networks demonstrated that non-infected controls displayed a balanced cross-talk between innate and adaptive immunity cells, represented by evenly distributed attributes, including positive and negative axis correlations. On the other hand, *T*. *cruzi* infected monkeys showed a clear shift toward a bimodal network profile with preferential circuit involving the adaptive immunity compartment, represented by moderate and strong positive correlation axes (Fig 3).

### System Biology Analysis of Innate Immunity Compartment Elected Monocytes and NK-Cell Phenotypes as the Most Promising Biomarkers with Putative Clinical Application

**Fig 4:**
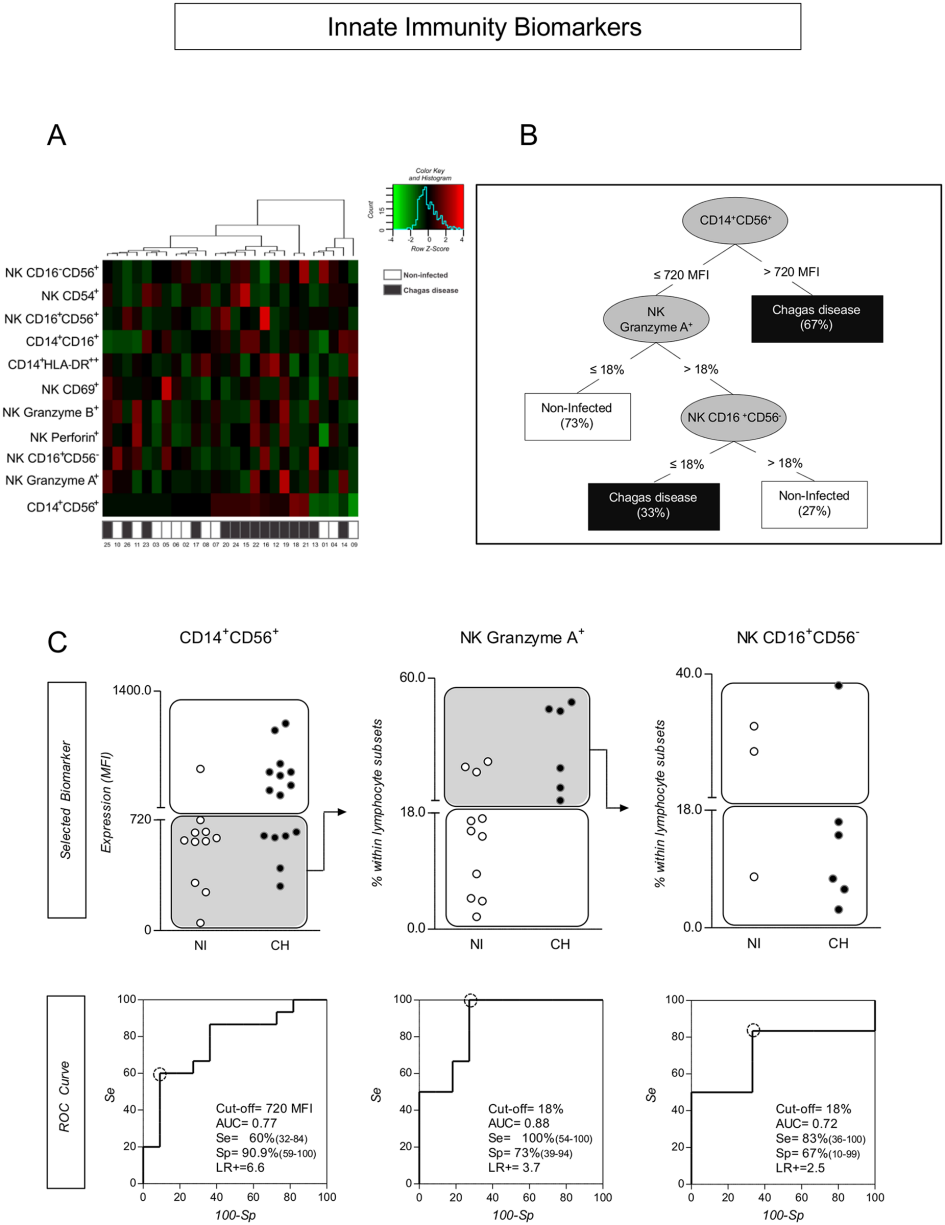
Systems biology strategy for analyzing innate immunity flow-cytometry data by heatmap and decision-tree analysis. (A) Bioinformatics tool applied for single-cell data mining using heatmap computational method to preprocess flow cytometry data and to identify the innate immunity cell attributes. (B) Decision tree analysis identifies “root” (CD14^+^CD56^+^) and “secondary” (NK Granzyme A^+^ and NK CD16^+^CD56^-^) cell attributes with higher accuracy to distinguish between non-human primates naturally infected with *T*. *cruzi* and non-infected controls. (C) Scatter distribution plots show the potential of selected biomarkers to discriminate infected from non-infected individuals. White rectangles indicate true positive (Chagas disease) and true negative (non-infected subjects) classifications. Gray rectangles indicate subjects that require the analysis of additional characteristics for accurate classification by the algorithm sequence proposed by the decision tree. (C) ROC curve analysis illustrating the cut-off points, the global accuracy (area under the curve–AUC) and performance indexes (sensitivity–Se, specificity–Sp and likelihood ratio–LR) for each selected biomarker.

To verify the profile of innate immunity associated with *T*. *cruzi* infection in primates, we constructed a matrix in a heat map representation (Fig 4A). Moreover, we carried out a decision tree classification in other to identify the innate immunity biomarkers most able to discriminate infected from control monkeys (Fig 4B). The heat map analysis of innate immunity clearly demonstrated the ability of CD14^+^CD56^+^ biomarker to cluster most infected monkeys apart from the uninfected controls (Fig 4A). This finding was further confirmed in the decision tree classification analysis that indicated this biomarker as the most relevant element, followed by NK Granzyme A^+^ cells and NK CD16^+^CD56^-^ cells which together represent the most promising set of innate immunity biomarkers with putative clinical application. The performance of these selected biomarkers was further investigated by scatter plot distribution and ROC curve analysis (Fig 4C). Our data demonstrated that CD14^+^CD56^+^/NK Granzyme A^+^/NK CD16^+^CD56^-^ cells together display a moderate global accuracy, ranging from 0.72 to 0.88 (Fig 4C). The decision tree displayed a mean accuracy of 0.54 by 10-fold cross validation, being more efficient in classifying monkeys as being infected (10 out of 15).

### Systems Biology Analysis of Adaptive Immunity Compartment Indicated T-Cell Activation Status and the Granzyme A Expression by CD8^+^ T-Cells as the Most Promising Biomarkers for Clinical Applications

**Fig 5:**
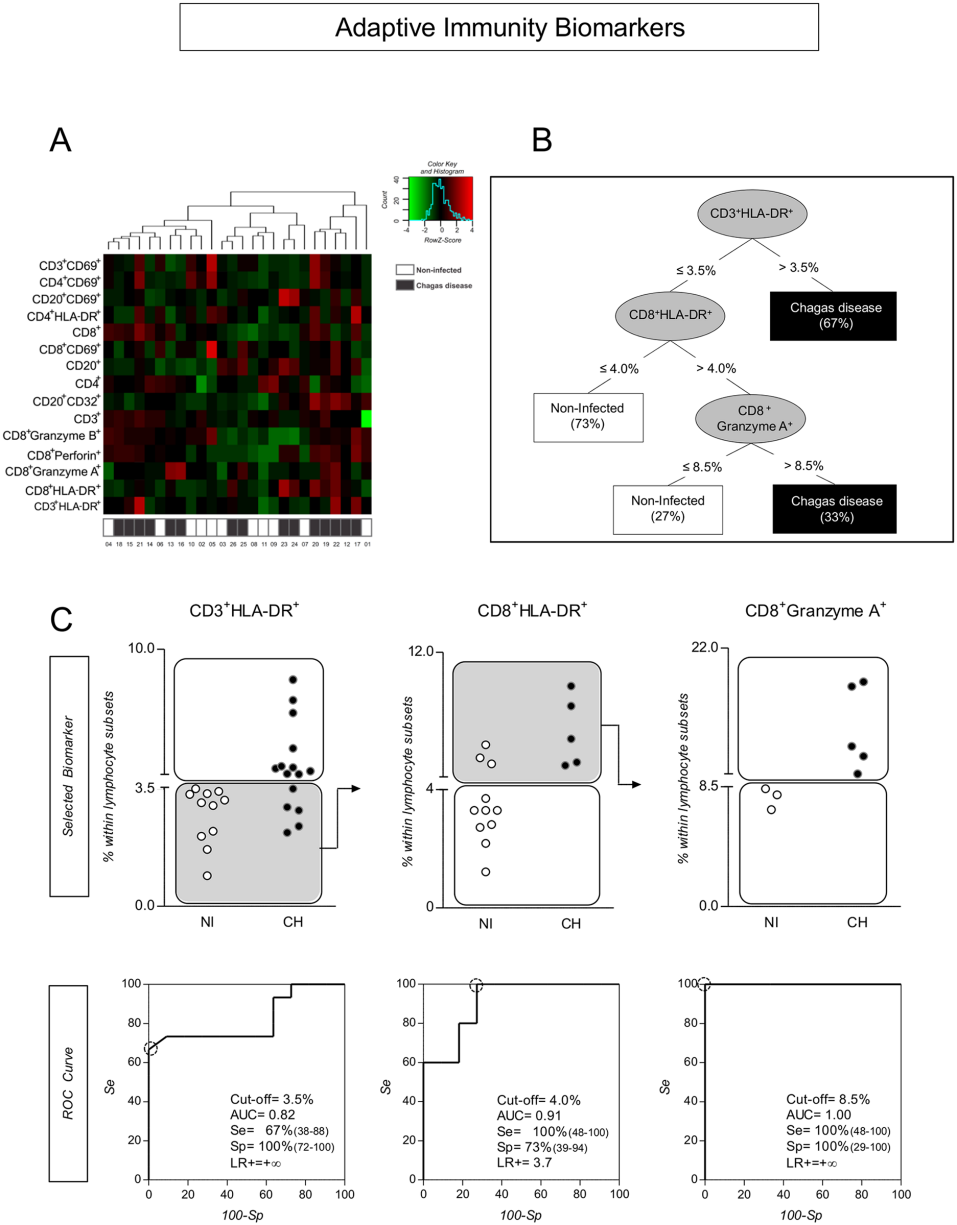
Systems biology strategy for analyzing adaptive immunity flow-cytometry data by heatmap and decision-tree analysis. (A) Bioinformatics tool applied for single-cell data mining using heatmap computational method to preprocess flow cytometry data and to identify the adaptive immunity cell attributes. (B) Decision tree analysis identifies “root” (CD3^+^HLA-DR^+^) and “secondary” (CD8^+^HLA-DR^+^ and CD8^+^ Granzyme A^+^) cell attributes with higher accuracy to distinguish between non-human primates naturally infected with *T*. *cruzi* and non-infected controls. (C) Scatter distribution plots show the potential of selected biomarkers to discriminate infected from non-infected individuals. White rectangles indicate true positive (Chagas disease) and true negative (non-infected subjects) classifications. Gray rectangles indicate subjects that require the analysis of additional characteristics for accurate classification by the algorithm sequence proposed by the decision tree. (C) ROC curve analysis illustrating the cut-off points, the global accuracy (area under the curve–AUC) and performance indexes (sensitivity–Se, specificity–Sp and likelihood ratio–LR) for each selected biomarker.

To verify the profile of adaptive immunity associated with *T*. *cruzi* infection in primates, we constructed a matrix in a heat map representation (Fig 5A). Additionally, we assembled a decision tree classification in order to identify the most promising adaptive immunity biomarkers able to distinguish infected monkeys from control monkeys (Fig 5B). The heat map analysis suggested that the HLA-DR activation marker, expressed by T-cells, especially by the CD8^+^ T-cell subset, is a reliable biomarker to identify infected monkeys (Fig 5A). This finding was further confirmed by the decision tree classification analysis that indicated that these biomarkers along with CD8^+^Granzyme A^+^ cells represent a good set of adaptive immunity biomarkers to support clinical investigations of *T*. *cruzi* infection of non-human primates. The performance of these selected biomarkers was further investigated by scatter plot distribution and ROC curve analysis (Fig 5C). Data analysis demonstrated that, together, CD3^+^HLA-DR^+^/CD8^+^HLA-DR^+^/ CD8^+^Granzyme A^+^ T-cells displayed a moderate global accuracy, ranging from 0.82 to 1.0 (Fig 5C), adjusted to 0.73 by 10-fold cross validation, for identifying infected subjects (12 out of 15) with low false-positive identification in the control group (4 out of 11).

## Discussion

In the present study, we have performed the phenotypic features of circulating leukocytes, focusing on the frequency of subsets and their activation status in cynomolgus macaques naturally infected with *T*. *cruzi*. The main findings revealed a similar pattern of immunological status likewise that observed in human indeterminate Chagas disease. The relevance of these results is to support the use of cynomolgus macaques in preclinical toxicology and pharmacology studies applied to development and testing of new drugs for Chagas disease.

It is well known that the immunological response plays a major role in the pathogenesis of Chagas disease [4]. To establish effective treatments, drug trials must be conducted in experimental models, prior to be administered to humans. Thus, it is important to validate an animal model that present similar immunological and clinical manifestations as those observed in humans. Indeed, non-human primates have demonstrated to be of great potential for such proposal, since they show similarities with human Chagas disease [10,11].

Macaques are important models for a remarkable diversity of human infectious diseases. Using these models, many studies have contributed novel insights into physiological and pathogenic mechanisms and also have revealed the involvement of distinct immunological events that can be used as comparative parameters in research on human diseases [10,12,19,20,21]. In fact, the study of experimentally induced or naturally occurring infectious diseases in non-human primates has enabled the establishment of valuable strategies for the development of improved vaccines, diagnostic tools, and therapeutic schemes for human illnesses [20]. In the present investigation, based on the occurrence of natural infections of *T*. *cruzi* in cynomolgus macaques, and the fact that the infected monkeys develop similar clinical sequelae [21], as well as equivalent histopathological patterns [12], to those of infected humans we have tested the hypothesis that these similarities are supported by equivalent immunological events in the two species. In addition to describing the immune mechanisms triggered by *T*. *cruzi* infection in these non-human primates, this study has presented a comprehensive overview of cells involved in innate and adaptive immunity using novel systems biology approaches to describe the cross-talk between immunological elements and to select candidate biomarkers relevant for clinical studies.

The immunophenotypic analysis of circulating leukocytes from cynomolgus macaques naturally infected with *T*. *cruzi* (CH) demonstrated that CH displayed increased expression of CD32^+^ and CD56^+^ in monocytes as compared to non-infected controls (NI). There is a general consensus that the innate immune response represents an important mechanism to control parasite replication during early and chronic Chagas disease [22]. The CD14^+^CD56^+^ monocyte subset has been related to human inflammatory chronic diseases [23] and is found on peripheral blood cells of healthy monkeys [24], but little is known about its role in humans and non-human primates infected with *T*. *cruzi*. These cells are capable of becoming more frequently positive for TNF-α cytokine, express higher levels of reactive oxygen species and FcγR (CD16^+^ and CD32^+^), are more efficient antigen presenting cells, and are a good generator of cytotoxic response [23,25]. These data suggest that CD14^+^CD56^+^ monocytes could represent an important cell population in determining Chagas disease progression, and further investigations of CD14^+^CD56^+^ monocytes are needed to better define their role in the immune response in *T*.*cruzi*-infected primates.

Our data also established that CH displayed higher frequency of cytotoxicity markers, represented by NK Granzyme A^+^ cells, by comparison to NI. In fact, it has been demonstrated by several studies that macrophages are efficiently activated by NK derived IFN-γ, which invokes nitric oxide production and controls parasite replication during *T*. *cruzi* infection [4,26]. Furthermore, the cytotoxic activity of NK cells could also contribute to control of parasitemia, through lysis of infected host cells or killing free parasites by contact-dependent exocytosis of lytic granules, independently from perforin [27]. These data are consistent with the hypothesis that higher NK cell cytotoxic activity could be important in helping to suppress the parasitemia to very low levels, resulting in avoidance of developing a strong acquired immune response involved with disease severity [16].

Although it is widely accepted that the adaptive immune response plays a critical role in ability to control Chagas disease progression in humans, in non-human primates its mechanisms remain unclear. Our findings showed that CH developed a similar pattern of T-lymphocytes as observed in human *T*. *cruzi* infection. In fact, higher expression of CD54 and HLA-DR by T-cells, especially within the CD8^+^ subset, along with outstanding expression of Granzyme A and Perforin was observed in CH, underscoring the enhanced cytotoxicity-linked pattern of CD8^+^ T-lymphocytes. In patients in the chronic phase of Chagas disease, a robust expansion of T-cell response to parasites has been clearly demonstrated [4]. Dutra et al. [28] showed a high frequency of activated T-cells in peripheral blood of indeterminate and cardiac patients, and further studies evaluated inflammatory infiltrate from heart tissue of cardiac patients, verifying a higher level of adhesion molecule expression by endothelial cells, as well as an increased frequency of Granzyme A^+^ CD8^+^ T-cells [29]. Previous to the findings reported here, we and other investigators have reported that non-human primates infected with *T*. *cruzi* develop chronic cardiomyopathy similar to that of humans. There are also reports of amastigote nests and parasite DNA with similar inflammatory infiltrates in heart tissue from *T*. *cruzi*-infected non-human primates [12,30,31,32], further supporting the premise that these animals are excellent experimental models for research on Chagas disease.

The analysis of the B-cell compartment revealed an expansion of these cells concomitant with up-regulated expression of Fc-γRII in CH. Previous studies have demonstrated that B-cells play an important role in systemic protection against *T*. *cruzi* through participation in the synthesis of anti-*T*. *cruzi* antibodies and in the maintenance of CD8^+^ memory T-cells, as well as in the determination of the T-cell cytokine functional pattern [33,34]. In regard to the role of CD32^+^ B-cells in human Chagas disease, it has been demonstrated that patients with asymptomatic clinical forms present lower levels of these modulatory CD32 surface marker with concomitant higher antibody levels, whereas cardiac patients presented baseline expression of CD32 by B-cells with lower antibody titers. A putative up-regulation of CD32 observed in B-cells from cynomolgus macaques could influence the degree of myocardiopathy found in these animals.

Several methods have been developed to draw networks of phenotypic aspects of the immune system in order to illustrate pathways and to describe clustering of cellular cross-talking relevant to understanding the dynamics of the immune system. Data mining of innate and adaptive immunity using the biomarker network approach revealed that whereas a balanced cross-talk between innate and adaptive immunity cells was observed in non-infected controls, CH primates demonstrated a clear shift toward a bimodal network profile with a preferential circuit involving the adaptive immunity compartment, supporting the existence of strong interaction between *T*. *cruzi* infection and the adaptive immunity cells, as observed in humans. The movement toward clustered nodes in the adaptive immunity compartment observed in CH is consistent with the chronic stage of *T*. *cruzi* infection in these animals. It is possible that the analysis of animals during early stages of infection would reveal a predominant involvement of innate immunity cells as is observed in humans with the early indeterminate form of Chagas disease [35].

Aiming to further identify cell phenotypes of innate and adaptive immunity compartments as promising biomarkers with putative clinical application, we have applied computational bioinformatics tools to explore the immunological findings observed in CH and control animals. Results from this approach suggest that the CD14^+^CD56^+^/NK Granzyme A^+^/NK CD16^+^CD56^-^ cells represented a good set of attributes from innate immunity to distinguish CH from NI. As for adaptive immunity, the CD3^+^HLA-DR^+^/CD8^+^HLA-DR^+^/CD8^+^Granzyme A^+^ T-cells were identified as the major subsets to discriminate CH from NI. The performance of these selected biomarkers was further validated by scatter plot distribution and ROC curve analysis. ROC curves were calculated to evaluate the capacity of these biomarkers to discriminate CH primates from the NI group. Moreover, they confirmed the superior performance of CD14^+^CD56^+^/NK Granzyme A^+^/NK CD16^+^CD56^-^ cells and CD3^+^HLA-DR^+^/CD8^+^HLA-DR^+^/ CD8^+^Granzyme A^+^ T-cells to distinguish CH animals from control animals. The data analysis demonstrated that these phenotypic attributes displayed a moderate global accuracy in identifying infected subjects, even after cross validation. In this context, CD14^+^CD56^+^ and CD3^+^HLA-DR^+^ were elected as the root attributes, as is consistent with the findings in human Chagas disease that highlight the robust role of macrophages and active T-cells as relevant biomarkers in the immune response triggered by *T*. *cruzi* infection [26]. Moreover, the secondary attribute branches, composed by NK-cells and CD8^+^ T-cells, are also in agreement with the recognized function of these cytotoxic cells in distinct processes during Chagas disease.

Importantly, our data demonstrated that the phenotypic features of circulating leukocytes from naturally infected non-human primates resembled the pattern in human Chagas disease. The current study may present some limitations concerning particularities of *T*. *cruzi* infection by genotype TcI that may differ from those triggered by infections with other genetic groups. Moreover, further analysis of putative immunological similarities related to cardiac Chagas disease may also be accomplished in future investigations. Nonetheless, the findings presented in the current investigation clearly demonstrated that, likewise humans, non-human primates with indeterminate *T*. *cruzi* infection develop an immunological profile involving both, innate and adaptive immune response that support the use of this experimental model for testing of new drugs for Chagas disease. Since cynomolgus macaques have an immunological response to *T*. *cruzi* very similar to that of humans, they also represent a useful experimental model for testing vaccines for Chagas disease.
